# Fungal Exposure and Low Levels of IL-10 in Patients with Sarcoidosis

**DOI:** 10.1155/2014/164565

**Published:** 2014-08-07

**Authors:** Marjeta Terčelj, Sanja Stopinšek, Alojz Ihan, Barbara Salobir, Saša Simčič, Ragnar Rylander

**Affiliations:** ^1^Department of Respiratory and Allergic Diseases, The University Medical Centre, Ljubljana, Slovenia; ^2^Institute of Microbiology and Immunology, Faculty of Medicine, University of Ljubljana, 1000 Ljubljana, Slovenia; ^3^Biofact Environmental Health Research Center, Björkåsvägen 21, 44391 Lerum, Sweden

## Abstract

*Background and Objectives*. Sarcoidosis is an inflammatory disease with increased levels of inflammatory cytokines. Previous studies have shown a relation between the degree of granuloma infiltration and serum cytokine levels, except for interleukin- (IL-) 10. The aim of the study was to further investigate the serum levels of IL-10 in patients with sarcoidosis and relate them to fungal exposure in terms of the amount of fungi in the air of their homes and *β*-glucan in bronchoalveolar lavage (BAL) fluid. *Methods*. Patients with sarcoidosis (*n* = 71) and healthy controls (*n* = 27) were enrolled. IL-10 was determined in serum. BAL was performed and the amount of *β*-glucan was measured. Domestic exposure to fungi was determined by measuring airborne *β*-N-acetylhexosaminidase (NAHA) in the bedrooms. *Results*. At high levels of fungal exposure (domestic fungal exposure and *β*-glucan in BAL), serum IL-10 values were lower than at low and intermediate exposure levels. *Conclusion*. The low serum IL-10 values at high fungal exposure suggest that fungal cell wall agents play a role in granuloma formation in sarcoidosis by inhibiting the secretion of the anti-inflammatory cytokine IL-10.

## 1. Introduction

Sarcoidosis is an inflammatory disease, often leading to granuloma formation [1, 2]. Several studies demonstrate that the amounts of inflammatory cytokines, particularly interleukin- (IL-) 10 and IL-12, are elevated in serum and in bronchoalveolar lavage fluid [3–6]. Previous studies have demonstrated that exposure to fungi is a risk factor for sarcoidosis [7–9]. One fungal cell wall agent (FCWA)—*β*-glucan—can induce different changes in the immune system and granulomas, depending on dose and means of administration (review in [10]). The formation of granuloma can be suppressed by IL-10 [11]. Chitin is another FCWA that can induce immune changes, depending on the size of the particles [12].

In* in vitro* studies on the reactivity of peripheral blood mononuclear cells (PBMC), particulate *β*-glucan was found to induce the secretion of TNF*α*, IL-6, IL-10, and IL-12 from PBMC [13] with a higher secretion from PBMC taken from patients with sarcoidosis [14].

A clinical study evaluated the relation between the extent of granuloma infiltration using an x-ray score and the amount of serum TNF*α*, IL-6, IL-10, IL-12, angiotensin converting enzyme (ACE), and chitotriosidase (CTO) [15]. There was a linear relationship for all inflammatory mediators and markers except for IL-10. For this cytokine, there was an initial increase with an increased X-ray score but the values were lower at the highest X-ray scores. This suggests that a blocking of the normal secretion of IL-10, due to inflammation, might be a mechanism related to the risk of granuloma formation in sarcoidosis. This observation prompted the present study which comprises an evaluation of serum levels of IL-10 in patients with sarcoidosis in relation to fungal exposure in terms of exposure to fungi at their home and the amount of *β*-glucan in BAL.

## 2. Material and Methods

### 2.1. Subjects

The subjects were recruited from the Department for Respiratory and Allergic Diseases at the University Medical Centre, Ljubljana, Slovenia, from July 2007 to October 2013. The department is one of the national centres for patients with sarcoidosis. For the diagnosis the ERS/ATS criteria [16] are used. The routine at the clinic is to make bronchoscopy with 5 to 10 transbronchial biopsies of lung parenchyma and needle aspiration of mediastinal lymph nodes. Bronchoalveolar lavage (BAL) is made and the CD4+/CD8+ ratio is determined. The presence of noncaseating granulomas is verified histologically. If a biopsy is not considered representative, the patient undergoes surgical pulmonary or lymph node biopsy. Aspiration is performed from the right, upper lobe for culturing pathogenic fungi and bacteria including tuberculosis. Most biopsies are stained (silver staining, Gomori) to identify the presence of fungal infection. IgA, IgM, and IgG antibodies against* Candida* spp. and* Aspergillus* spp. are determined as well as mannan antigen in blood. Diagnostic BAL and sputum on subjects with sarcoidosis demonstrated an absence of pathogenic fungi and TB.

This study comprises newly diagnosed cases of pulmonary sarcoidosis (*n* = 71) and healthy subjects (*n* = 26) were examined. All subjects were nonsmokers. The study was approved by the Ethical Committee at the University Medical Centre, Ljubljana (198/05/04), and written, informed consent was obtained.

### 2.2. Clinical Assessments

IL-10 was determined in serum using a commercial ELISA kit (Milenia Biotec, Germany, and Thermo Scientific, USA) and expressed as mmol/mL. Chitotriosidase (CTO) activity in serum was determined using 22 *μ*M 4-methylumbelliferyl-*β*-D-N,N′,N′′-triacetylchitotriosiose (Sigma) in citrate phosphate buffer (pH 5.2) and expressed as nmol/h/mL [17, 18].

### 2.3. Analysis of *β*-Glucan

Samples of the BAL fluid were mixed with 20 mL of a solution containing 0.15 mol/L KOH, 0.3 mol/L KCl, and 0.1% polybrene and incubated at 37°C for 10 minutes. For the analysis of *β*-glucan, a commercially available method based on the reactivity of a Limulus extract was used. The BAL preparation was diluted in a protein blocking buffer (Biodispersing agent, Charles River, Charleston, SC, USA), kept in boiling water for two minutes, and further diluted in endotoxin free water (LAL, Charles River). Thereafter, 25 *μ*L was added to each of the four wells in a plate preprepared with a Limulus reagent specific for *β*-glucan and read in an automatic analyser (Endosafe PTS, Charles River). The lower limit for detection is 1 pg/mL. Samples yielding a readout value of <100 pg/mL were given the uniform value of 90 to save on reagents. For ethical reasons BAL was only performed in nine control subjects.

### 2.4. Fungal Exposure at Home

The exposure to fungi in the homes was determined by analysing the amount of airborne *β*-N-acetylhexosaminidase (NAHA) as a marker of fungal cell biomass [19, 20]. Air samples (around 2000 L) were taken in the subject's bedroom using a filter and a fluorogenic enzyme substrate (4-methylumbelliferyl N-acetyl-*β*-D-glucosaminide, Mycometer A/S, Copenhagen, Denmark) was added to the filter. After an incubation period of around 30 minutes, set by room temperature, a developer was added, and the fluorescence of the liquid was read in a fluorometer (Picofluor, Turner Designs, Sunnyvale, CA, USA). The units read were divided by 10 to diminish methodological scatter and expressed as NAHA Units/m^3^.

### 2.5. Statistical Analysis

Values in the different groups were calculated using SPSS W7 and were expressed as mean and standard error of the mean (SEM). Differences between groups were evaluated using the *t*-test or Fisher's exact test. A *P* value of ≤0.05 was considered statistically significant.

## 3. Results 

### 3.1. Characteristics of Test Subjects

Basic characteristics of the subjects are shown in Table 1.

The clinical values are typical for newly diagnosed sarcoidosis. There were four cases of skin sarcoidosis.

Serum IL-10 values are reported in Table 2.

There were no significant differences in mean values between controls and sarcoidosis. The maximum value was higher among subjects with sarcoidosis. In controls all *β*-glucan values were less than 90 pg/mL and NAHA values were less than 30 U/m^3^ except for one outlier (76).

**Figure 1:**
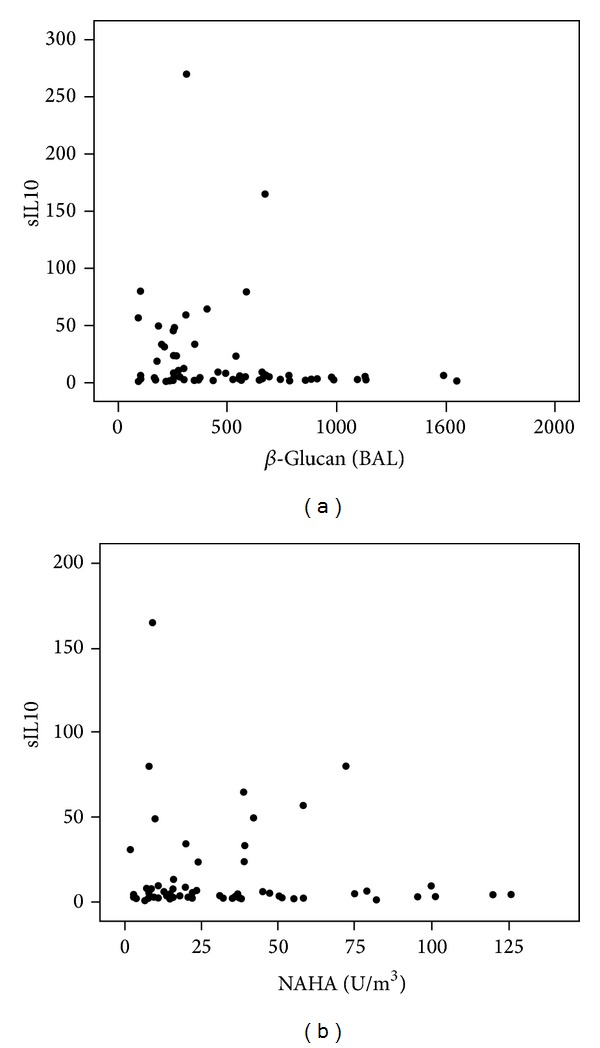
(a) Serum IL-10 in relation to the amount of *β*-glucan (BAL) pg/mL (*n* = 70) and (b) NAHA in homes (*n* = 60) of subjects with sarcoidosis.

Figure 1 illustrates serum levels of IL-10 among subjects with sarcoidosis in relation to *β*-glucan in BAL.

At higher amounts of *β*-glucan in BAL and NAHA in the homes, reflecting a higher exposure to fungi, there were no high levels of IL-10. At a break-off point of 750 pg/mL *β*-glucan, the mean IL-10 value in the lower *β*-glucan value group was 23.5/5.7 as compared to 3.8/0.5 for the higher value group (*P* = 0.026, Mann-Whitney's test). At a break-off point of NAHA of 75, the corresponding IL-10 values were 20.6/8.9, *n* = 65, and 5.6/1.5, *n* = 7 (*P* = 0.0001, Fisher's exact test).

## 4. Discussion

The major findings from the study are that the serum values of IL-10 were low at higher exposure to fungi, as illustrated by the domestic exposure and the amount *β*-glucan in BAL.

There are some limitations to the study. The number of subjects is fairly small, particularly in the high fungal exposure group. Measurements of NAHA were made in the homes but significant exposures could also have been present at the workplace. BAL for *β*-glucan determination could not be performed in all subjects because of ethical reasons.

A previous study has shown that values of IL-10 were lower at higher levels of CTO, a marker of disease severity [15]. In this study a similar decrease was present in relation to the fungal exposure at home. This suggests that there is a depression of IL-10 secretory capacity at higher exposure levels. Almost all cells of both the innate and adaptive arms of the immune system can express IL-10 including dendritic cells, macrophages, mast cells, natural killer cells, eosinophils, neutrophils, and T regulatory cells [21, 22]. This cytokine is one of the most important anti-inflammatory and immune suppressive cytokines. It affects the vascular system through inhibition of leukocyte-endothelial cell interaction and inhibition of proinflammatory cytokine and chemokine production by macrophages and lymphocytes [23]. In an animal model low levels of IL-10 were a predisposing factor for chronic fibrosis [24]. Of particular interest in sarcoidosis is that IL-10 can inhibit an experimental granulomatous inflammation [11] and that corticosteroids, which are usually used to treat the disease, have the capacity to enhance IL-10 production [25].

This opens up a new possible mechanism to explain the relation between fungal exposure and sarcoidosis [9]. It is tempting to speculate that the development of granulomas in sarcoidosis is initiated by an inflammation induced by inhaled *β*-glucan and that this inflammation at higher exposure levels depresses an important defence mechanism in terms of IL-10. Further work is required to assess this hypothesis. Although the focus in this context has been on fungi, it cannot be excluded that other microbial agents may induce similar reactions in terms of depression of the cellular capacity to secrete IL-10. To assess the importance of such agents, for example, Mycobacteria, studies on exposure levels as well as the effect on cellular mechanisms are required.

## 5. Conclusions

At a high environmental exposure to fungi, the level of serum IL-10 among subjects with sarcoidosis was low. This suggests that *β*-glucan could play a role in the granuloma formation in sarcoidosis by blocking a defence system in terms of IL-10 secretion. At high levels of fungal exposure patients with sarcoidosis have a depressed secretion of the anti-inflammatory cytokine IL-10. This suggests a new mechanism for the development of granulomas in sarcoidosis.

## Figures and Tables

**Table 1:** Basic characteristics of subjects with sarcoidosis. Mean and (SEM).

Parameter	Controls	Sarcoidosis	
*n*	26	71	
Age years	57.6 (5.2)	47.4 (1.4)	
Females %	63	49	
NAHA U/m^3^	19.0 (5.0)	34.1 (3.9)	*P* < 0.001
*Β*-glucan pg/mL	122.5 (16.1)	474.5 (38.6)	*P* < 0.001
CD4/CD8 ratio		7.5 (0.7)	
CTO nmol/h/mL		775 (68)	
sACE *μ*Kat/L		0.49 (0.03)	

**Table 2:** IL-10 values (pg/mL) in serum of subjects with sarcoidosis and controls. Mean, SEM and range.

Subjects	*n*	Mean	SEM	Minimum	Maximum	*P*
Controls	26	10.3	2.8	1	63	
Sarcoidosis	71	16.6	3.7	1	270	NS
